# Supplementary material for: Studying the dimensions of m-interactivity and customers’ engagement in m-commerce applications
Source: PeerJ Comput Sci. 2023 May 22;9:e1392. doi: 10.7717/peerj-cs.1392 (PMC10280500; doi:10.7717/peerj-cs.1392)
Supplement: Supplemental Information 3 — The questionnaire was distributed in this Google Form (Arabic Language) among five hundred Saudi citizens randomly. The Partial least squares (PLS) were implemented to analyse the data to test the proposed hypotheses and get the rest of the results. [file peerj-cs-09-1392-s003.pdf]

دراسة تأثير أبعاد تفاعل الأجهزة المحمولة على مشاركة العملاء في تطبيقات التجارة الإلكترونية عبر الهاتف المحمول في المملكة العربية السعودية

* Required

تهدف هذه الدراسة إلى دراسة تأثير أبعاد تفاعل الأجهزة المحمولة على مشاركة العملاء في تطبيقات التجارة الإلكترونية عبر الهاتف المحمول في المملكة العربية السعودية . أيضا ، فإنها تهدف إلى اختبار تأثير تفاعل العملاء على ولائهم تجاه تطبيقات التجارة الإلكترونية عبر الهاتف المحمول.

نأمل المشاركة في هذا الاستبيان وستساعد إجاباتك الباحثين على تحليل البيانات و الحصول على نتائج بحثية جيدة . نقدر وقتك في الإجابة على جميع الأسئلة. سيستغرق إكمال هذا الاستبيان حوالي 8 دقائق. يرجى قراءة التعليمات واستكمال الاستبيان. هذا الاستبيان مجهول تماما(لا يتطلب معلومات شخصية) . مشاركتك في هذه الدراسة ليست إلزامية ويمكنك التوقف عن مشاركتك في أي وقت دون إبداء أي أسباب. ستتم حماية معلوماتك واستخدامها فقط لتحقيق متطلبات البحث. تم الحصول على تصريح أخلاقي من جامعة شقراء في كلية الحاسب الآلي الرقم المرجعي لأخلاقيات البحث البشري (Ref: Ethics Appl.220302022) والذي يهدف للتأكد من إجراء هذا البحث بالطريقة الصحيحة. سوف تكون موافقتك على المشاركة في هذا البحث ضمنية من خلال قراءة المعلومات واستكمال الاستبيان وإعادته.

من فضلك لا تتردد في الاتصال إذا كان لديك أي أسئلة وشكرا جزيلا لك مقدما على مشاركتك.

مع الشكر والتقدير
د. عبدالرحمن الغامدي
alghamdia@su.edu.sa

1. الجنس *

Mark only one oval.

- ( ) ذكر
- ( ) أنثى

2. العمر *

Mark only one oval.

- أقل من 18 سنة
- من 18 سنة الى 40 سنة
- من 41 سنة الى 60 سنة
- أكبر من 60 سنة

3. المؤهلات *

Mark only one oval.

- الابتدائية
- الثانوية
- دبلوم
- بكالوريوس
- الدراسات العليا.

4. المهنة *

Mark only one oval.

- طالب
- أعمل في مؤسسة حكومية
- أعمل في القطاع الخاص
- رجل أعمال
- آخرى.

5. منذ متى وأنت تستخدم تطبيقات التجارة الالكترونية عبر الهاتف المحمول ؟ *

Mark only one oval.

- ( ) لم أستخدم تطبيقات التجارة الالكترونية عبر الهاتف المحمول مطلقًا
- ( ) أقل من عام
- ( ) من 1 سنة إلى أقل من 2 سنة
- ( ) من 2 إلى أقل من 3 سنوات
- ( ) من 3 سنوات أو أكثر.

Active control التحكم النشط

6. أستطيع التنقل بسهولة عبر تطبيقات التجارة الإلكترونية عبر الهاتف المحمول *

Mark only one oval.

- ( ) أوافق بشدة
- ( ) أوافق
- ( ) محايد
- ( ) لا أوافق
- ( ) لا أوافق بشدة

7. أثناء تصفحي لتطبيقات التجارة الإلكترونية عبر الهاتف المحمول، تكون أفعالي بناء على تجاربي السابقة مع تطبيقات التجارة الإلكترونية عبر الهاتف المحمول *

Mark only one oval.

- ( ) أوافق بشدة
- ( ) أوافق
- ( ) محايد
- ( ) لا أوافق
- ( ) لا أوافق بشدة

8. أشعر أني أستطيع التحكم بقدر كبير في تجربتي مع تطبيقات التجارة الإلكترونية عبر الهاتف المحمول *

Mark only one oval.

- أوافق بشدة
- أوافق
- محايد
- لا أوافق
- لا أوافق بشدة

9. يمكنني التحكم بشكل كبير في تجربتي أثناء تصفح تطبيقات التجارة الإلكترونية عبر الهاتف المحمول. *

Mark only one oval.

- أوافق بشدة
- أوافق
- محايد
- لا أوافق
- لا أوافق بشدة

10. أستطيع التحكم بشكل كامل في عدد زياراتي لتطبيقات التجارة الإلكترونية عبر الهاتف المحمول *

Mark only one oval.

- أوافق بشدة
- أوافق
- محايد
- لا أوافق
- لا أوافق بشدة

11. أشعر أني أستطيع التحكم بقدر كبير في استخدام تطبيقات التجارة الإلكترونية عبر الهاتف المحمول *

Mark only one oval.

- أوافق بشدة
- أوافق
- محايد
- لا أوافق
- لا أوافق بشدة

12. يمكنني اختيار ما أريد رؤيته بحرية عند استخدامي تطبيقات التجارة الإلكترونية عبر الهاتف المحمول. *

Mark only one oval.

- أوافق بشدة
- أوافق
- محايد
- لا أوافق
- لا أوافق بشدة

إضفاء الطابع الشخصي Personalization

13. تتيح لي تطبيقات التجارة الإلكترونية عبر الهاتف المحمول طلب المنتجات أو الخدمات المصممة خصيصًا لي *

Mark only one oval.

- أوافق بشدة
- أوافق
- محايد
- لا أوافق
- لا أوافق بشدة

14. الإعلانات والعروض الترويجية التي ترسلها تطبيقات التجارة الإلكترونية عبر الهاتف المحمول إليّ مصممة خصيصًا لتناسب وضعي *

Mark only one oval.

- أوافق بشدة
- أوافق
- محايد
- لا أوافق
- لا أوافق بشدة

15. تطبيقات التجارة الإلكترونية عبر الهاتف المحمول تجعلني أشعر أنني عميل فريد *

Mark only one oval.

- أوافق بشدة
- أوافق
- محايد
- لا أوافق
- لا أوافق بشدة

16. يتم تقديم العروض المخصصة من خلال تطبيقات التجارة الإلكترونية عبر الهاتف المحمول *

Mark only one oval.

- أوافق بشدة
- أوافق
- محايد
- لا أوافق
- لا أوافق بشدة

17. يتم إرسال الرسائل المخصصة عن طريق تطبيقات التجارة الإلكترونية عبر الهاتف المحمول *

Mark only one oval.

- أوافق بشدة
- أوافق
- محايد
- لا أوافق
- لا أوافق بشدة

18. توفر تطبيقات التجارة الإلكترونية عبر الهاتف المحمول إمكانيات اجراء بحثًا مخصصًا عن المعلومات *

Mark only one oval.

- أوافق بشدة
- أوافق
- محايد
- لا أوافق
- لا أوافق بشدة

اتصال في كل مكان Ubiquitous connectivity

19. يمكنني الوصول إلى تطبيقات التجارة الإلكترونية عبر الهاتف المحمول في أي وقت للحصول على المعلومات أو الخدمة الضرورية *

Mark only one oval.

- أوافق بشدة
- أوافق
- محايد
- لا أوافق
- لا أوافق بشدة

20. يمكنني استخدام تطبيقات التجارة الإلكترونية عبر الهاتف المحمول "في أي مكان" و "في أي وقت" عند الحاجة *

Mark only one oval.

- أوافق بشدة
- أوافق
- محايد
- لا أوافق
- لا أوافق بشدة

21. تتيح لي تطبيقات التجارة الإلكترونية عبر الهاتف المحمول طلب المنتجات أو الخدمات في أي مكان وفي أي وقت *

Mark only one oval.

- أوافق بشدة
- أوافق
- محايد
- لا أوافق
- لا أوافق بشدة

22. يمكنني الوصول إلى تطبيقات التجارة الإلكترونية عبر الهاتف المحمول في أي مكان للحصول على المعلومات أو الخدمات الضرورية *

Mark only one oval.

- أوافق بشدة
- أوافق
- محايد
- لا أوافق
- لا أوافق بشدة

23. أشعر أنني متصل دائمًا بتطبيقات التجارة الإلكترونية عبر الهاتف المحمول *

Mark only one oval.

- أوافق بشدة
- أوافق
- محايد
- لا أوافق
- لا أوافق بشدة

24. يمكنني التواصل بسهولة مع تطبيقات التجارة الإلكترونية عبر الهاتف المحمول بغض النظر عن الزمان والمكان *

Mark only one oval.

- أوافق بشدة
- أوافق
- محايد
- لا أوافق
- لا أوافق بشدة

الترابط Connectedness

25. يشارك عملاء تطبيقات التجارة الإلكترونية عبر الهاتف المحمول خبراتهم حول المنتج أو الخدمة مع عملاء آخرين *

Mark only one oval.

- أوافق بشدة
- أوافق
- محايد
- لا أوافق
- لا أوافق بشدة

26. يستفيد عملاء تطبيقات التجارة الإلكترونية عبر الهاتف المحمول من المجتمع الذي ترعاه هذه التطبيقات *

Mark only one oval.

- أوافق بشدة
- أوافق
- محايد
- لا أوافق
- لا أوافق بشدة

27. يشترك عملاء تطبيقات التجارة الإلكترونية عبر الهاتف المحمول في رابطة مشتركة مع أعضاء آخرين في مجتمع برعاية هذه التطبيقات *

Mark only one oval.

- أوافق بشدة
- أوافق
- محايد
- لا أوافق
- لا أوافق بشدة

28. كوني جزءًا من مجتمع تطبيقات التجارة الإلكترونية عبر الهاتف المحمول يجعلني أشعر بأنني أكثر ارتباطًا بالعلامة التجارية التي أحبها *

Mark only one oval.

- أوافق بشدة
- أوافق
- محايد
- لا أوافق
- لا أوافق بشدة

29. كوني جزءًا من مجتمع تطبيقات التجارة الإلكترونية عبر الهاتف المحمول يجعلني أشعر بأنني أكثر ارتباطًا بمستهلكين آخرين من نفس العلامات التجارية التي أحبها *

Mark only one oval.

- أوافق بشدة
- أوافق
- محايد
- لا أوافق
- لا أوافق بشدة

إستجابة Responsiveness

30. تطبيقات التجارة الإلكترونية عبر الهاتف المحمول لديها القدرة على الرد على أسئلتي المحددة ذات الصلة *

Mark only one oval.

- أوافق بشدة
- أوافق
- محايد
- لا أوافق
- لا أوافق بشدة

31. تسهل تطبيقات التجارة الإلكترونية عبر الهاتف المحمول الاتصال ثنائي الاتجاه بين العملاء والشركات *

Mark only one oval.

- أوافق بشدة
- أوافق
- محايد
- لا أوافق
- لا أوافق بشدة

32. المعلومات التي تظهر بعد تعاملي مع تطبيقات التجارة الإلكترونية عبر الهاتف المحمول تلبي توقعاتي *

Mark only one oval.

- أوافق بشدة
- أوافق
- محايد
- لا أوافق
- لا أوافق بشدة

33. المعلومات التي تظهر بعد تعاملي مع تطبيقات التجارة الإلكترونية عبر الهاتف المحمول تكون مناسبة *

Mark only one oval.

- أوافق بشدة
- أوافق
- محايد
- لا أوافق
- لا أوافق بشدة

34. عندما أستخدم تطبيقات التجارة الإلكترونية عبر الهاتف المحمول ، يمكنني دائمًا الاعتماد على تلقي الكثير من الردود على أسئلتي وتعليقاتي *

Mark only one oval.

- أوافق بشدة
- أوافق
- محايد
- لا أوافق
- لا أوافق بشدة

التزامن Synchronicity

35. تعالج تطبيقات التجارة الإلكترونية عبر الهاتف المحمول مدخلاتي بسرعة كبيرة *

Mark only one oval.

- أوافق بشدة
- أوافق
- محايد
- لا أوافق
- لا أوافق بشدة

36. الحصول على المعلومات من تطبيقات التجارة الإلكترونية عبر الهاتف المحمول سريع جدًا *

Mark only one oval.

- أوافق بشدة
- أوافق
- محايد
- لا أوافق
- لا أوافق بشدة

37. يمكنني الحصول على المعلومات التي أريدها دون أي تأخير *

Mark only one oval.

- أوافق بشدة
- أوافق
- محايد
- لا أوافق
- لا أوافق بشدة

38. أشعر أنني أحصل على معلومات فورية *

Mark only one oval.

- أوافق بشدة
- أوافق
- محايد
- لا أوافق
- لا أوافق بشدة

39. يبدو أن تطبيقات التجارة الإلكترونية عبر الهاتف المحمول سريعة جدًا في الاستجابة لطلباتي *

Mark only one oval.

- أوافق بشدة
- أوافق
- محايد
- لا أوافق
- لا أوافق بشدة

40. عندما أنقر على الروابط الموجودة في تطبيقات التجارة الإلكترونية عبر الهاتف المحمول ، أشعر أنني أحصل على معلومات فورية *

Mark only one oval.

- أوافق بشدة
- أوافق
- محايد
- لا أوافق
- لا أوافق بشدة

تطوير مشترك Co-developing

41. أتواصل بشكل استباقي مع تطبيقات التجارة الإلكترونية عبر الهاتف المحمول بشأن المشكلات المحتملة المتعلقة بالخدمة *

Mark only one oval.

- أوافق بشدة
- أوافق
- محايد
- لا أوافق
- لا أوافق بشدة

42. أقدم اقتراحات بناءة لتطبيقات التجارة الإلكترونية عبر الهاتف المحمول حول كيفية تحسين خدماتها *

Mark only one oval.

- أوافق بشدة
- أوافق
- محايد
- لا أوافق
- لا أوافق بشدة

43. أسمح لتطبيقات التجارة الإلكترونية عبر الهاتف المحمول بمعرفة الطرق التي يمكن أن تخدم احتياجاتي بشكل أفضل *

Mark only one oval.

- أوافق بشدة
- أوافق
- محايد
- لا أوافق
- لا أوافق بشدة

التأثير Influencing

44. أقول أشياء إيجابية للآخرين عن تطبيقات التجارة الإلكترونية عبر الهاتف المحمول وعن موظفيه *

Mark only one oval.

- أوافق بشدة
- أوافق
- محايد
- لا أوافق
- لا أوافق بشدة

45. أوصي الآخرين باستخدام تطبيقات التجارة الإلكترونية عبر الهاتف المحمول. *

Mark only one oval.

- أوافق بشدة
- أوافق
- محايد
- لا أوافق
- لا أوافق بشدة

46. أشجع الأصدقاء والأقارب على استخدام تطبيقات التجارة الإلكترونية عبر الهاتف المحمول في المستقبل *

Mark only one oval.

- أوافق بشدة
- أوافق
- محايد
- لا أوافق
- لا أوافق بشدة

Augmenting جعل (شيء) أعظم من خلال الإضافة إليه ؛ زيادة

47. أنشر صورًا لنشاطي باستخدام تطبيقات التجارة الإلكترونية عبر الهاتف المحمول على وسائل التواصل الاجتماعي. *

Mark only one oval.

- أوافق بشدة
- أوافق
- محايد
- لا أوافق
- لا أوافق بشدة

48. أود أن أكتب مدونات عن تجربتي الإيجابية مع تطبيقات التجارة الإلكترونية عبر الهاتف المحمول. *

Mark only one oval.

- أوافق بشدة
- أوافق
- محايد
- لا أوافق
- لا أوافق بشدة

49. توفر تطبيقات التجارة الإلكترونية عبر الهاتف المحمول فرصًا لمشاركة تجربتي مع الآخرين عبر وسائل التواصل الاجتماعي. *

Mark only one oval.

- أوافق بشدة
- أوافق
- محايد
- لا أوافق
- لا أوافق بشدة

50. أشارك في إعادة توجيه العروض الترويجية التي تقدمها تطبيقات التجارة الإلكترونية عبر الهاتف المحمول إلى الآخرين. *

Mark only one oval.

- أوافق بشدة
- أوافق
- محايد
- لا أوافق
- لا أوافق بشدة

التعبئة Mobilizing

51. أساعد العملاء الآخرين إذا كانوا بحاجة إلى مساعدتي *

Mark only one oval.

- أوافق بشدة
- أوافق
- محايد
- لا أوافق
- لا أوافق بشدة

52. أقدم النصيحة للعملاء الآخرين فيما يتعلق بخدمات تطبيقات التجارة الإلكترونية عبر الهاتف المحمول. *

Mark only one oval.

- أوافق بشدة
- أوافق
- محايد
- لا أوافق
- لا أوافق بشدة

53. أقوم بتعليم العملاء الآخرين استخدام الخدمات بشكل صحيح. *

Mark only one oval.

- أوافق بشدة
- أوافق
- محايد
- لا أوافق
- لا أوافق بشدة

54. أساعد العملاء الآخرين إذا كان لديهم مشاكل. *

Mark only one oval.

- أوافق بشدة
- أوافق
- محايد
- لا أوافق
- لا أوافق بشدة

55. أنا على استعداد للعمل على حماية سمعة تطبيقات التجارة الإلكترونية عبر الهاتف المحمول *

Mark only one oval.

- أوافق بشدة
- أوافق
- محايد
- لا أوافق
- لا أوافق بشدة

56. أنا على استعداد لتوضيح سوء فهم العملاء الآخرين أو الغرباء فيما يتعلق بتطبيقات التجارة الإلكترونية عبر الهاتف المحمول *

Mark only one oval.

- أوافق بشدة
- أوافق
- محايد
- لا أوافق
- لا أوافق بشدة

الولاء Loyalty

57. سأوصي باستخدام تطبيقات التجارة الإلكترونية عبر الهاتف المحمول لأشخاص آخرين *

Mark only one oval.

- أوافق بشدة
- أوافق
- محايد
- لا أوافق
- لا أوافق بشدة

58. أنوي الاستمرار في استخدام تطبيقات التجارة الإلكترونية عبر الهاتف المحمول *

Mark only one oval.

- أوافق بشدة
- أوافق
- محايد
- لا أوافق
- لا أوافق بشدة

59. أفضل استخدام تطبيقات التجارة الإلكترونية عبر الهاتف المحمول على قنوات التسوق الأخرى *

Mark only one oval.

- أوافق بشدة
- أوافق
- محايد
- لا أوافق
- لا أوافق بشدة

60. سأختار تطبيقات التجارة الإلكترونية عبر الهاتف المحمول حتى إذا كانت خيارات التسوق البديلة متاحة *

Mark only one oval.

- أوافق بشدة
- أوافق
- محايد
- لا أوافق
- لا أوافق بشدة

This content is neither created nor endorsed by Google.

Google Forms
